# Thunder and lightning—a report on firework-associated acoustic trauma at New Year 2021/2022

**DOI:** 10.1007/s00106-022-01260-z

**Published:** 2023-02-27

**Authors:** Veronika Flockerzi, Bernhard Schick, German Acoustic Fireworks-associated Traumata Study (GAFATS) Group, Justus Ilgner, Gerhard Hesse, Peter Jecker, Herbert Eichwald, Steffen Dommerich, Andreas O. H. Gerstner, Stephanie Hoppe, Jörg Ebmeyer, Jan Peter Thomas, Friedemann Papst, Joachim Hornung, Stephan Lang, Andreas Knopf, Philipp Dost, Christoph Arens, Christian Wrobel, Jörg Langer, Adrian Münscher, Thomas Lenarz, Olcay Cem Bulut, Matti Hein, Johanna Inhestern, Veronika Flockerzi, Bernhard Schick, Alessandro Bozzato, Philippe Federspil, Petra Ambrosch, Sandra Schmidt, O. Ebeling, Efastathios Papatsoutsos, Claudia Scherl, Sandra Schmidt, Boris A. Stuck, Benedikt Hofauer, Basel Al Kadah, Klaus Bumm, Martin C. Jäckel, Gregor Hilger, Birgit Muschal, Sven Becker, Theo Evers, Alessandro Bozzato

**Affiliations:** Klinik für Hals‑, Nasen- und Ohrenheilkunde, Universitätsklinikum des Saarlandes, 66421 Homburg, Germany

**Keywords:** Environment, Fireworks, Noise-induced hearing loss, Tinnitus, Epidemiology

## Abstract

**Background:**

This cross-sectional study aimed to assess the frequency and type of firework-associated acoustic trauma occurring in Germany on New Year’s Eve 2021, despite the ban on firework sales due to the COVID-19 pandemic.

**Materials and methods:**

The survey period lasted 7 days, from 28 December 2021 to 03 January 2022. A questionnaire inquired date, type and treatment of trauma, sex, and age of the patient, and whether the trauma occurred when lighting or watching fireworks. Hearing impairment was classified according to the World Health Organization (WHO grades 0 to 4), and concomitant tinnitus, vertigo, or other injuries were recorded. The questionnaire was sent to the otorhinolaryngology departments of 171 hospitals in Germany.

**Results:**

Of 37 otorhinolaryngology departments, 16 reported no and 21 reported 50 patients with firework-associated acoustic trauma. Mean age was 29 ± 16 years and 41 of 50 patients were males. Of these 50 patients, 22 presented without and 28 with hearing loss, 32 reported tinnitus and 3 vertigo; 20 patients were injured when lighting fireworks and 30 when watching. Hearing impairment was classified as 14 × WHO grade 0, 5 × WHO grade 1, 4 × WHO grade 2, 2 × WHO grade 3, and 3 × WHO grade 4. Inpatient treatment was received by 8 patients and 11 suffered from concomitant burn injuries.

**Conclusion:**

Despite the sales ban, some firework-associated acoustic traumas occurred at New Year 2021/2022 in Germany. Some instances led to hospitalization, but an even higher number of unreported cases can be assumed. This study can serve as a baseline for further annual surveys to raise the awareness of the danger of seemingly harmless fireworks for the individual.

**Supplementary Information:**

The online version of this article 10.1007/s00106-022-01260-z contains additional material: Questionnaire: German Acoustic Firework-associated Traumata Study (GAFATS) 2021.

Fireworks are supposed to bring joy and drive away bad spirits to welcome the New Year and have their origins in the Asian tradition of heating bamboo, which results in firecracking sounds [8]. Much has changed since their origin around 200 B.C. [8], but the desired sound effect remains the same.

## Economic significance of fireworks

The revenue of the pyrotechnics industry in Germany has almost continuously increased over the past 20 years from 102 million euro in 2000 to 137 million euro in 2016 and 2017 [18]. A significant decline to 20 million euro was observed in 2020 due to the first ban on the sale of fireworks [18]. The main reasons for this ban were the reduction of crowds so as to decrease the likelihood of the transmission of COVID-19 and “to prevent injuries when fireworks are lit on New Year’s Eve in order to relieve the strain on hospitals and emergency departments, which are already very busy due to the Corona pandemic” [3]. There is active lobbying by the pyrotechnics industry association to allow private fireworks again in the future. In a press release from December 2021 [19], they reported that New Year’s fireworks would have a low harm potential and that only 5% of all hospital treatments on New Year’s Eve were due to fireworks. Furthermore, they cited a question to the Bavarian State Parliament [1] regarding the busyness of police and rescue services on New Year’s Eve 2019/2020. In Bavaria, there were only 25 people injured by lighting fireworks—however, information about the age of those having been affected as well as the type and severity of injuries was missing. In addition, there was no description reflecting the situation in Germany. The observer is thus confronted with several questions: Are injuries caused by fireworks on New Year’s Eve a relevant factor in the healthcare system with possible relevance for the uninvolved individual? Are these injuries avoidable and if so, how?

## Summary of studies to date

Many studies have investigated the noise of exploding fireworks at various distances [8, 9, 11, 14]. One study concluded that even at a distance of 6 m from the firecracker, noise levels may exceed 110 decibels sound pressure level (dB SPL, [8]). The shorter the distance to the fireworks, the higher the sound level to which the individual is exposed: Up to 160 dB SPL may be reached at a distance of 2 m [14]—this corresponds to the sound level of a fired pistol [13]. Professional fireworks even go beyond this and can reach sound levels of more than 190 dB SPL even at short distances [9]. Noise levels of 90–130 dB SPL have been reported to induce production of reactive oxygen and nitrogen species and other free radicals that may lead to cochlear damage, while noise above 130 dB SPL induces mechanical injury to the inner ear [8]. Thus, the acoustic effects of fireworks affect not only those who actively light the fireworks, but also the spectators, and therefore acoustic trauma can also occur in apparently uninvolved persons. In the literature these individuals persons are often referred to as “bystanders” [5, 6].

Other medical disciplines, such as the German Ophthalmological Society, have analyzed this issue recently and have established a nationwide registry for the survey of firework-associated eye injuries with the aim of providing reliable data for a discussion on protective measures, including a possible ban on private fireworks [5].

The last survey of firework-associated acoustic trauma in Germany was published in 2002 and estimated an incidence of 10/100,000 on New Year’s Eve 1999 [11]. The ban on the sales of fireworks in 2020 and 2021 in the context of the COVID-19 pandemic in Germany was taken as an opportunity to establish a registry of firework-associated acoustic trauma with a baseline on New Year’s Eve 2021 in collaboration with the German Otorhinolaryngology Society. In addition to the discussion outlined at the beginning, we wanted to collect and to provide up-to-date data from the perspective of otorhinolaryngologists.

The purpose of this study was to document the number and severity of firework-associated acoustic trauma that occurred in Germany despite the fireworks sales ban in 2021 as a starting point for further annual comparisons.

## Methods

This retrospective cross-sectional study followed the principles of the Declaration of Helsinki and is based on a questionnaire that was sent to the otorhinolaryngology departments of 42 university hospitals and of 129 city hospitals in November 2021 (Supplemental data). The local ethics committee of Saarland (*Ethikkommission bei der Ärztekammer des Saarlandes*) was informed and they decided that this study was exempt because it does not contain any individual-related data. The survey period lasted 7 days around New Year’s Eve 2021 from 28 December 2021 to 3 January 2022 and the German otorhinolaryngology departments were asked to provide their patient data anonymously and to respond to the questionnaire even if there was no patient presenting with firework-associated acoustic trauma during this period. The questionnaire inquired about the (1) date, (2) type, and (3) treatment of the acoustic trauma; the (4) sex and (5) age of the patients; and (6) whether the trauma occurred when lighting or watching fireworks. Hearing impairment was classified according to the World Health Organization grades of 0 to 4 [7, 20]. Concomitant complaints such as tinnitus, vertigo, or other injuries were additionally recorded. Descriptive statistical analyses were performed.

## Results

**Fig. 1:**
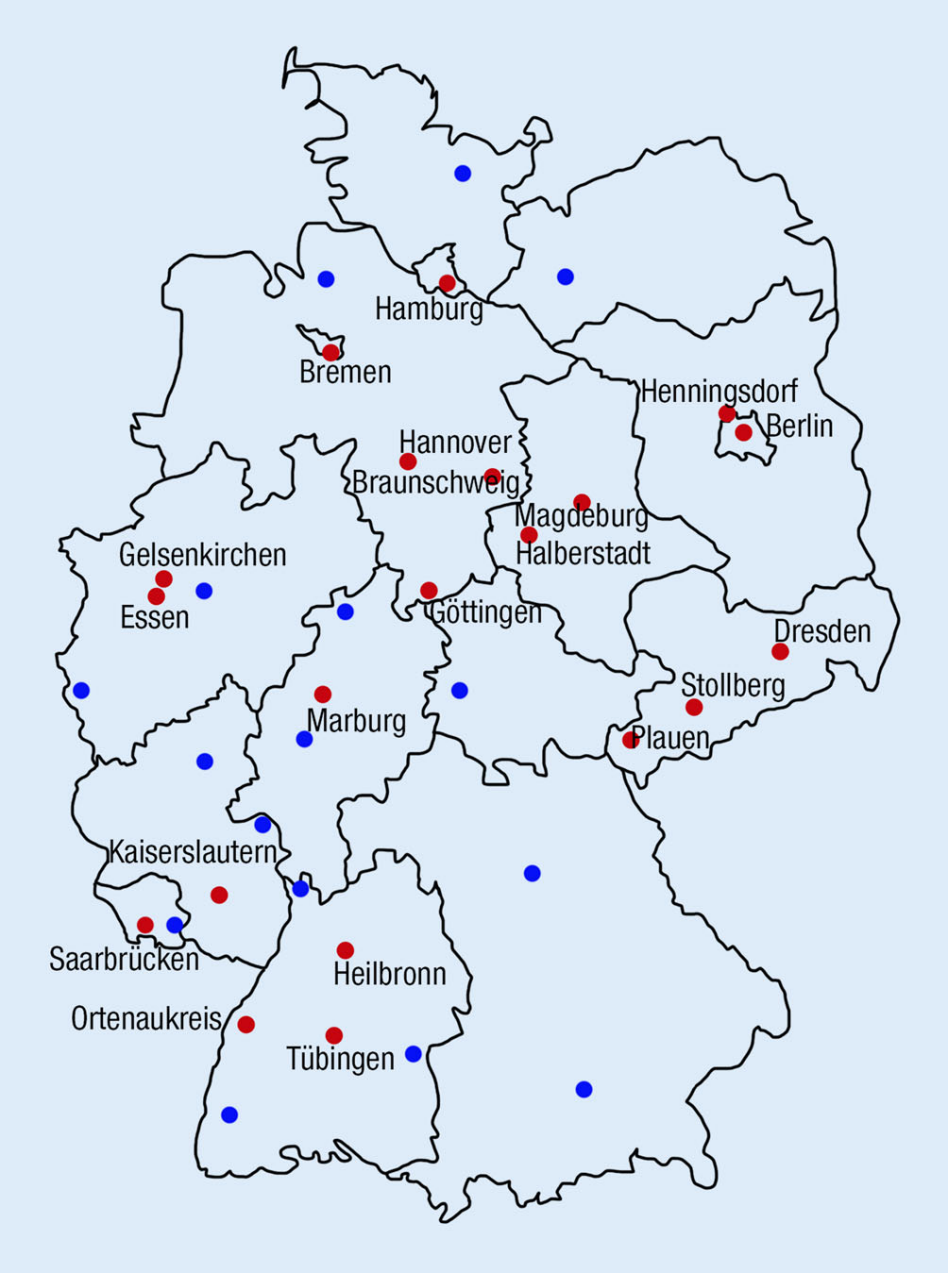
Participating hospitals. Map of Germany showing the reporting hospitals. *Red*: Patient reports (in Berlin, two hospitals reported patients). *Blue*: No patient presentations during the survey period. The participating hospitals are distributed throughout Germany

**Fig. 2:**
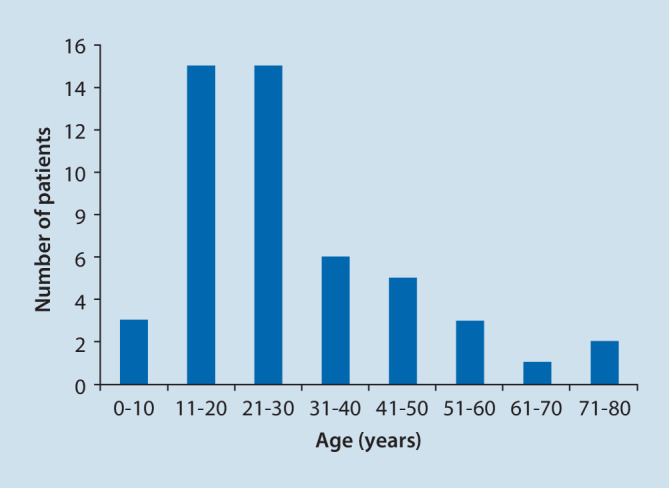
Age distribution of patients by decade. Overall, 60% of patients were between 11 and 30 years old; 12 of them were beyond the legal age. Decades—0–10: *n* = 3; 11–20: *n* = 15; 21–30: *n* = 15; 31–40: *n* = 6; 41–50: *n* = 5; 51–60: *n* = 3; 61–70: *n* = 1; 71–80: *n* = 2

Of 171 German otorhinolaryngology departments, 37 responded and provided their data on firework-associated acoustic trauma on New Year’s Eve 2021. In 15 of these 37 departments, no patient presented with such trauma during the survey period. The remaining 22 departments reported 50 patients with firework-associated acoustic trauma. Participating hospitals were distributed throughout Germany without any discernible pattern (Fig. 1). The mean age of these patients was 29 ± 16 years with an age range of 7–73 years. Overall, 60% of those affected were between 11 and 30 years of age. A total of 12 persons were below the legal age (Fig. 2). Out of these, six were injured while lighting fireworks, two were bystanders. The cause of injury was not indicated for two more people in this group. There was a male preponderance with 41 male patients (mean age 28 ± 16 years, 82%) and nine female patients (mean age 32 ± 18 years, 18%). In total, 20 patients (40%) were injured when lighting fireworks by themselves and 30 (60%) were injured as bystanders.

**Fig. 3:**
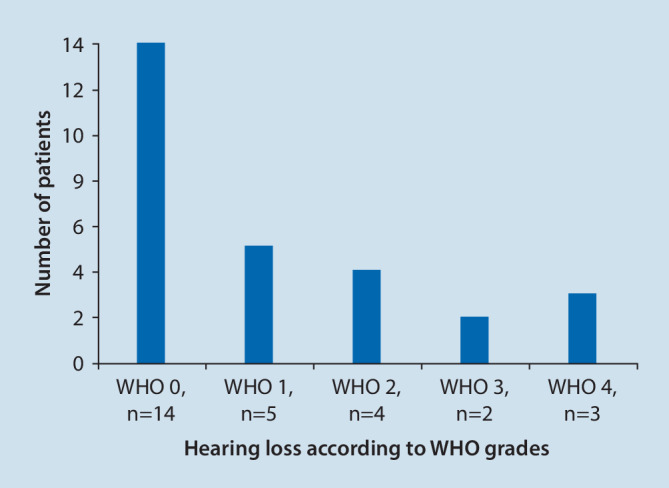
Grading of hearing impairment according to the World Health Organization Grades 0 to 4. Half of the patients (*n* = 14) classified with hearing impairment of WHO grade 0, and a smaller number of patients with more severe hearing impairment grades (WHO grade 1: *n* = 5, WHO grade 2: *n* = 4, WHO grade 3: *n* = 2, WHO grade 4: *n* = 3)

Of these 50 patients, 22 presented without and 28 with hearing loss, 32 reported tinnitus, and three patients reported vertigo without verifiable nystagmus. Among the patients reporting tinnitus or hearing impairment, 13 reported isolated tinnitus, nine reported isolated hearing impairment, and 19 reported a combination of tinnitus and hearing impairment. Hearing impairment was classified according to the World Health Organization of grades 0 to 4 ([7, 20], Fig. 3). Half of the patients with hearing impairment were classified as WHO grade 0 (*n* = 14), five patients as WHO grade 1, four patients as WHO grade 2, two patients as WHO grade 3, and three patients as WHO grade 4.

A total of 11 patients suffered concomitant burn injuries mostly in the face or on their hands. Among them, two patients were bystanders and one was not further specified. Eight of them were injured when actively lighting fireworks and six of these patients were treated as inpatients.

**Fig. 4:**
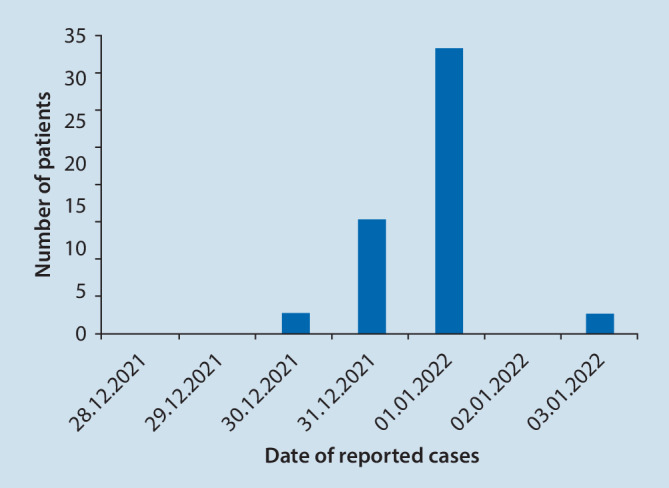
Distribution of reported patients by date of injury. More than 90% of patients presented on 31 December 2021 and 1 January 2022 (*n* = 33, 66% on 01/01/2022; *n* = 13, 26% on 12/31/2021). Two patients were reported on 12/30/2021 and two on 01/03/2022

The highest number of injuries was reported on 1 January 2022 (*n* = 33). There were 13 cases of trauma reported on New Year’s Eve. On 30 December 2021 and on 3 January 2022, there were two cases of trauma reported (Fig. 4).

Two cases of new traumatic tympanic membrane perforation were reported. One of them suffered from bilateral tympanic membrane perforation due to chronic otitis media prior to the trauma.

## Discussion

This retrospective cross-sectional study aimed to summarize firework-associated acoustic trauma around New Year’s Eve 2021 in Germany as assessed by a questionnaire that was sent to the otorhinolaryngology departments of 42 university hospitals and of 129 city hospitals (in total 171 hospitals).

### Patient characteristics and organs affected

The survey revealed that the typical patient with firework-associated acoustic trauma was a male patient, 28 ± 16 years of age, who lit the fireworks by himself. A total of 60% of those affected were between 11 and 30 years old, while 24% were below the legal age. A previous study conducted in Germany in 1999 also found a male preponderance with firework-associated acoustic trauma occurring 3 times more often in males than in females at an average age of 19 years [11]. The 2020 Fireworks Annual Report of the United States of America Consumer Product Safety Commission stated that the highest risk for firework-associated injury is for people between 20 and 24 years of age and that after the hand and fingers, ears are the second most commonly affected by firework-associated trauma along with face and head injuries [17]. Similar results were found in an analysis by the National Electronic Injury Surveillance System in the United States that registered 2641 firework-related injuries during a period from 2008 to 2017: In this analysis, ears were the third most commonly affected (11%) by firework-related injuries after facial (62%) and head (13%) injuries [16]. Strikingly, the authors found the highest probability of being injured in children up to 12 years of age (40%), followed by adults over 22 years of age (33%) and adolescents from 13 to 21 years of age (27%, [16]). Similar to this finding and to the numbers reported by the ophthalmological *Feuerwerks-Verletzungen-Studiengruppe* [5] concerning only eye injuries, children and adolescents (defined as persons below legal age, 40% in [5]) are overrepresented in our study with 24% compared to 16.7% in the normal population [15]. The current study also found the majority of patients (60%) with firework-associated acoustic trauma to be bystanders. The numbers shown here are comparable to those in ophthalmological studies [5, 6]. A possible explanation might be that bystanders are less aware of the danger of approaching fireworks than persons who light fireworks themselves and thus cannot take protective measures (e.g., distance, hearing protection).

### Noise injury

It has been reported that firework noise may reach 160 dB SPL [8, 9, 14], which corresponds to the sound level of a fired pistol [13]. One may suppose that adequate hearing protection would protect against hearing damage from firearms, but even this does not apply in general. A 10-year longitudinal study in police officers revealed a 75% cervical vestibular-evoked myogenic potential abnormality after monthly target shooting practice for over 10 years despite wearing ear plugs [21]. The authors attributed this damage to a transmission of vibration injury to the saccule [21]. These results make firework-associated acoustic trauma even more complicated and treacherous in two aspects: First, the combination of noise and vibration may potentiate the adverse effects of noise on the cochlea [12, 21]. Second, the noise and hazard associated with it are underestimated the shorter its duration is, which typically applies for the short-term effects of fireworks [10]. In general, the greatest absolute hearing recovery is observed in the most severely damaged patients. At the same time, permanent damage is also greatest in these patients [4].

### Prevention

Regarding the questions outlined at the beginning: Are injuries caused by fireworks on New Year’s Eve a relevant factor in healthcare system with possible relevance for the uninvolved individual? Are these injuries avoidable and if so, how? Based on the data presented in this manuscript, these questions can be answered as follows in our opinion: Injuries caused by fireworks are also relevant for the individual, since they disproportionately often affect particularly vulnerable groups: children and uninvolved bystanders [5, 16]. Although access to category two pyrotechnics is restricted to adults—thus representing a restriction for children [2]—half of the children and adolescents reported here were injured while actively lighting fireworks. In our opinion, one way to avoid injuries in the future is to restrict the access to fireworks further, especially for children, and also to inform parents of the specific dangers and existing legal regulations. Spectators should also pay attention to the dangers of fireworks.

The present study collected the number and type of firework-associated acoustic trauma cases in Germany despite the sales ban. Therefore, a limited number of reports was to be expected. We agree with the ophthalmological *Feuerwerks-Verletzungs-Studiengruppe* in Germany that the dynamics of occurring injuries should be monitored over several years in order to conclusively assess changes in use and the effect of legal regulations—such as the current sales ban [5].

### Limitations of the study

There remain some limitations. First, only otorhinolaryngology departments of hospitals were contacted. This could be a limitation, as patients may also have presented to and been treated by an otorhinolaryngologist in private practice and were not referred to the clinic. However, it is also likely that many practices were closed during the study period, which would mitigate this presumed error. Nevertheless, a relevant number of unreported cases must be assumed because patients sometimes believe that the hearing loss is temporary [11].

A clear limitation is certainly the low response rate of the questionnaire among German otorhinolaryngology departments, which should be markedly improved in future surveys. Consequently, one aim of this publication is also to increase awareness of the problem of firework-associated acoustic trauma among German otorhinolaryngologists and to thereby increase the response rate in future surveys. Moreover, we are working on the creation of an online questionnaire to enable “paperless” data collection within the next 2 years.

Additionally, it has been reported that it is more likely for patients to encounter the firework impact on the ipsilateral side when they use either their left or right hand to light the firecracker [22]. Therefore, future surveys should ask not only about the side of the injury, but also whether the patient is right- or left-handed.

The effect of the fireworks sales ban in 2021 will become apparent and measurable by comparing it with future surveys of periods without a sales ban.

## Practical conclusion

- A small number of reported cases of firework-associated acoustic trauma around New Year’s Eve 2021 were reported in Germany despite the fireworks sales ban because of the COVID-19 pandemic.
- This study is not without limitations, but it may serve as a baseline for further annual surveys to raise awareness of the danger of seemingly harmless fireworks for (the ears of) the individual.
- Further data on firework-associated acoustic trauma will be collected in the years to come.
- The survey is planned to be distributed in Germany in the newsletter of the German Otorhinolaryngology Society.

## Supplementary Information

Questionnaire: German Acoustic Firework-associated Traumata Study (GAFATS) 2021

## Declarations

For this article no studies with human participants or animals were performed by any of the authors. All studies mentioned were in accordance with the ethical standards indicated in each case.
