# Supplementary material for: Thunder and lightning—a report on firework-associated acoustic trauma at New Year 2021/2022
Source: HNO. 2023 Feb 27;71(Suppl 1):44–9. doi: 10.1007/s00106-022-01260-z (PMC9969936; doi:10.1007/s00106-022-01260-z)
Supplement: Supplementary file 1 — Questionnaire: German Acoustic Firework-associated Traumata Study (GAFATS) 2021 [file 106_2022_1260_MOESM1_ESM.pdf]

Questionnaire:

German Acoustic Firework-associated Traumata Study (GAFATS) 2021

Date of trauma		Lighting firework by themselves
Age	Years	
Sex	m: f:	Spectators

Injuries

**Hearing impairment**	No	Yes				
		Sensorineural	Conductive	Combined		
		WHO Grade	0	≤ 25db		
			1	26-40db		
			2	41-60db		
			3	61-80db		
			4	≥ 81db		
		Treatment				
**Tinnitus**	No	Yes				
**Vertigo**	No	Yes				
		Nystagm	No	Yes		
				right	left	vertical
**Other injuries**	No	Yes				
		Eardrum perforation	No	Yes		

Treatment

Outpatient	Inpatient
Medication (e.g. steroids, pentoxifyllin, tebonin…)	Surgery (e.g. infusion, tympanoplasty…)

Remarks

